# Dataset on phenolic profile of seven wheat genotypes along maturation

**DOI:** 10.1016/j.dib.2018.09.111

**Published:** 2018-10-04

**Authors:** Talita Pimenta do Nascimento, Millena Cristina Barros Santos, Luciana Ribeiro da Silva Lima, Fabiana Ramos Nascimento, Luiz Claudio Cameron, Mariana Simões Larraz Ferreira

**Affiliations:** a Laboratory of Bioactives, Food and Nutrition Graduate Program, Federal University of State of Rio de Janeiro, Av. Pasteur, 296, Urca, 22290-240 Rio de Janeiro, Brazil; b Department of Food Science, Nutrition School, UNIRIO, Brazil; c Center of Innovation in Mass Spectrometry, Laboratory of Protein Biochemistry, UNIRIO, Brazil

## Abstract

This article contains data on phenolic-profiling of seven wheat genotypes along maturation (softy, milky, physiological maturity and mature). This supplementary data is related to research “Metabolomic approach for characterization of phenolic compounds in different wheat genotypes during grain development” (Santos et al., 2018). Briefly, free and bound phenolic compounds were extracted with 80% ethanol (v/v) and hydrolysis processes, respectively. The aliquots resultants were separated by ultra-performance liquid chromatography (UPLC) and analyzed by quadrupole time-of-flight mass spectrometry (QTOF). Data were acquired using a multiplexed MS/MS acquisition with alternating low and high energy acquisition (MS^E^). The phenolic compounds with their respective abundances are showed here through characterization table and multivariate analysis (hierarchical cluster analysis—HCA—and principal component analysis—PCA).

**Specifications table**

**Table:** 

Subject area	Food science
More specific subject area	Metabolomic
Type of data	Table and graphs
How data was acquired	Phenolics measurements were obtained using ultra-performance liquid chromatography (UPLC) coupled with quadrupole time-of-flight mass spectrometry operating in MS^E^ mode (QTOF-MS^E^).
Data format	Data analyzed with Progenesis QI, MassLynx, Metaboanalyst and XLSTAT.
Experimental factors	Free and bound phenolic compounds from different wheat genotypes harvested along grain development were extracted with ethanol and hydrolysis processes, respectively.
Experimental features	Separation and identification of phenolic compounds in wheat genotypes, using UPLC-QTOF-MS^E^.
Data source location	Wheat ears harvested in Passo Fundo, Brazil (Latitude S 28°09′33″, Longitude W 52°18′23″) and mass spectrometry data acquired in Rio de Janeiro, Brazil.
Data accessibility	Data provided in the article are accessible to the public.

**Value of the data**

• The data provide details on profile of phenolic compounds in wheat genotypes along grain maturation.
• Method and data make available information on how determinate the profile of phenolic compounds by UPLC- QTOF-MS^E^.
• Data processing by multivariate statistical tools is provided to reveal the most significant discriminatory compounds.
• Chemometric tools can help to distinguish grain maturation phases and also genotypes for breeding purposes.

## 1 Data

**Fig. 1:**
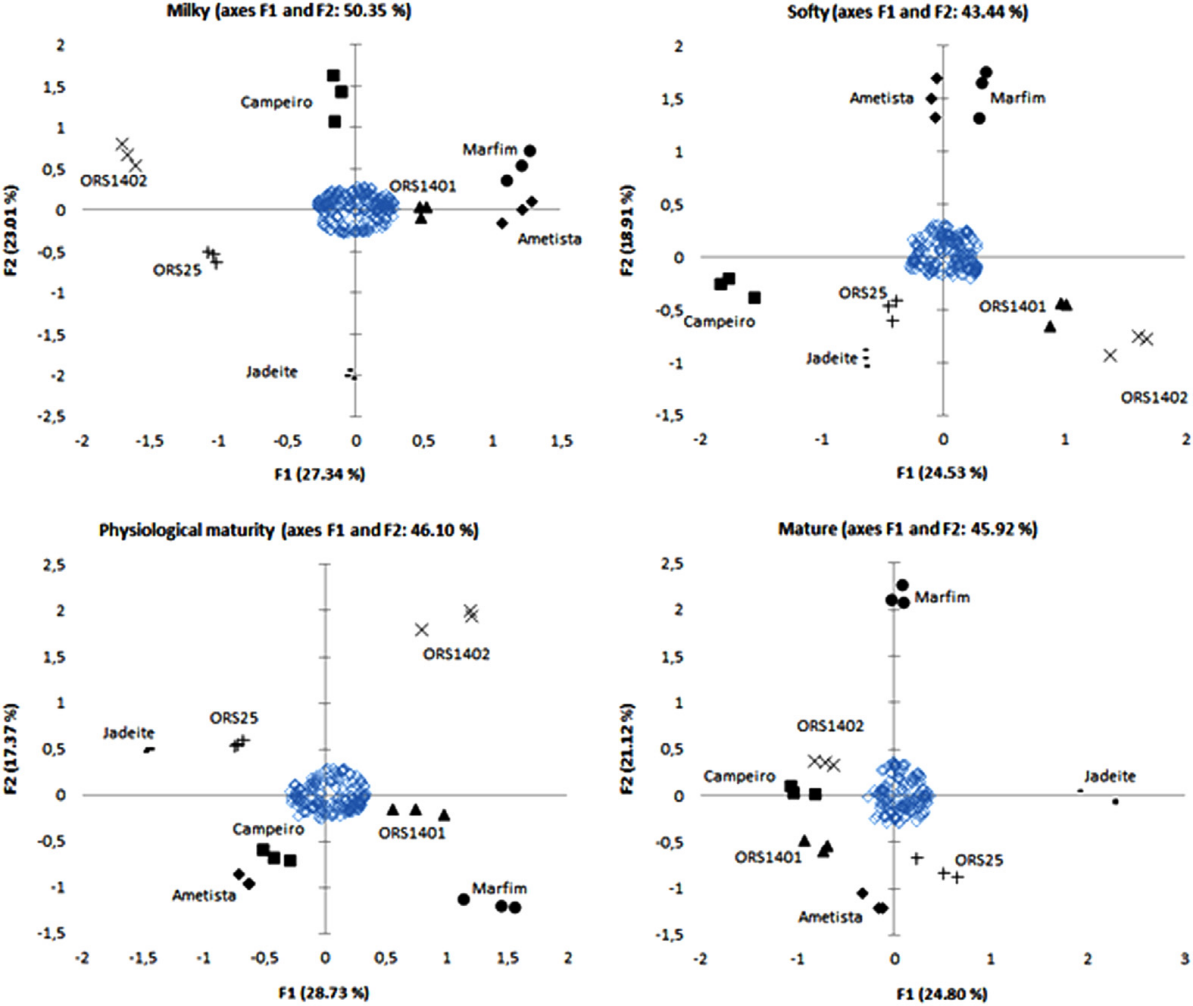
Principal component analysis (PCA) of the identified phenolics of immature (milky and softy stages) and mature (physiological maturity and mature stages) wheat genotypes.

**Fig. 2:**
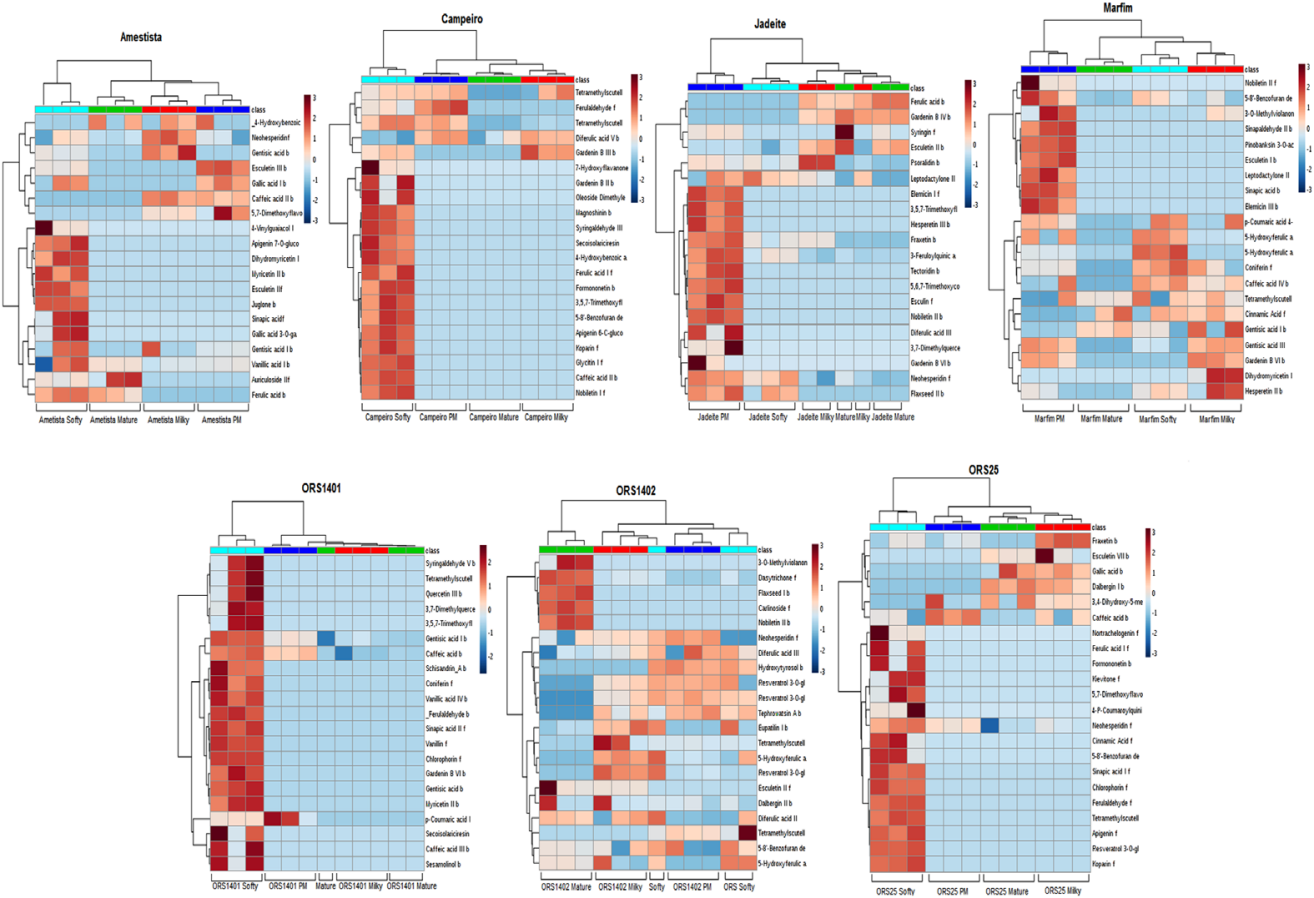
Hierarchical cluster analysis (HCA) and heatmap of the bound (b) and free (f) phenolics, which showed maximum variance (eigenvectors) in the wheat genotypes. Eigenvectors of the correlation matrix are described in the Appendix 4.

The data contains information on profile of phenolic compounds of wheat genotypes along grain maturation. Globally, a total of 370 compounds of free and bound phenolics were characterized in Appendix 1, including isomeric forms. In addition, the Appendix A, Appendix A depict the chromatograms of wheat genotypes in free and bound extractors in the different stages of maturation. Utilizing XLSTAT and Metaboanalyst, multivariated data of wheat genotypes were analyzed by principal component analysis—PCA (Fig. 1) and hierarchical cluster analysis—HCA (Fig. 2).

## 2 Experimental design, material and methods

### 2.1 Design

Wheat (*Triticum aestivum*) ears from seven cultivars (Campeiro, ORS25, ORS1401, ORS1402, Marfim, Jadeite and Ametista) harvested during four stages of grain development were defined according to the moisture content of the grains: milky (60%), softy (45%), physiological maturity (33%) and mature (12%), as described in [1]. Immature wheat grains from different genotypes were chosen to follow the evolution of the identified compounds during the grain development, and to assess the influence of genetic diversity on the composition of secondary metabolites, mainly of phenolic compounds.

### 2.2 Sample extraction

Free and bound phenolic compounds (PC) from ground whole grains were extracted with 80% ethanol (v/v) and hydrolysis processes (alkaline and acidity) as described in [1].

### 2.3 Chromatography and mass spectrometry

Phenolic extracts were injected in triplicate into the system UPLC Acquity (Waters Co., Milford, MA) coupled to the Xevo G2-S Q-Tof (Waters Co., Manchester, UK) equipped with an electrospray ionization source (ESI). A UPLC HSS T3 C18 column (100 mm × 2.1 mm, 1.8 μm particle diameter) (Waters) was used at 30 °C and gradient with 0.3% formic acid and 5 mM ammonium formate (mobile phase A) and acetonitrile containing 0.3% formic acid (mobile phase B) as described in [1].

### 2.4 Data analysis

The dataset was analyzed and processing using Progenesis QI v.2.1 (NonLinear Dynamics, Waters Co), XLSTAT (Addinsoft, Paris, France) and Metaboanalyst 3.0 web server [2].

## Figures and Tables

**Supplementary Fig. S1:**
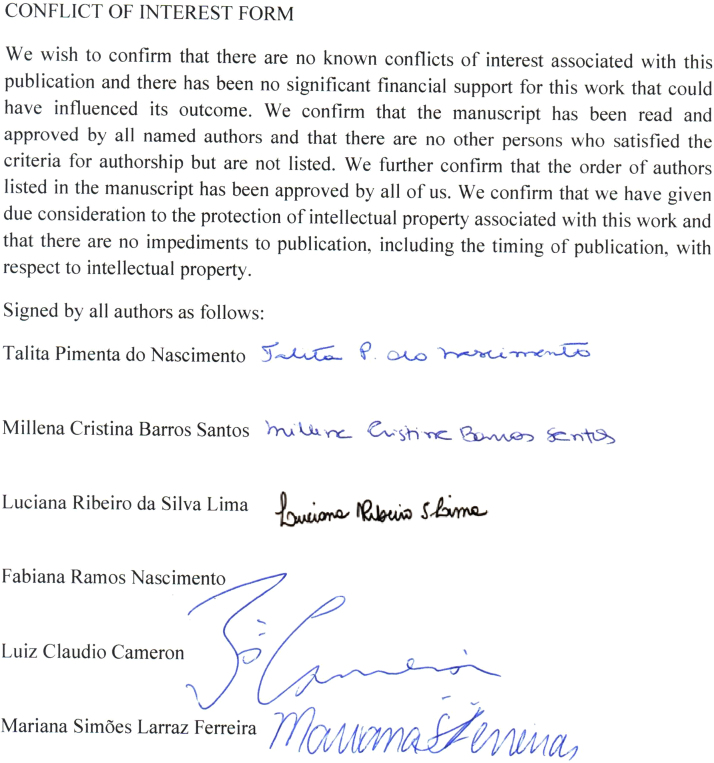


## Appendix A Supplementary material

Supplementary material Appendix 1: Phenolic compounds detected and characterized in different genotypes of wheat grains along maturation.

.

Supplementary material Appendix 2: Chromatograms of wheat genotypes in free extractors in different stages of maturation. Chromatogram of Jadeite genotype was shown in [1].

.

Supplementary material Appendix 3: Chromatograms of wheat genotypes in bound extractors in different stages of maturation. Chromatogram of Jadeite genotype was shown in [1].

.

Supplementary material Appendix 4: Eigenvectors of the correlation matrix corresponding to phenolics dataset of genotypes along maturation.
