# Supplementary material for: Dataset on phenolic profile of seven wheat genotypes along maturation
Source: Data Brief. 2018 Oct 4;21:284–8. doi: 10.1016/j.dib.2018.09.111 (PMC6197959; doi:10.1016/j.dib.2018.09.111)
Supplement: Supplementary file 1 — Supplementary material Appendix 1: Phenolic compounds detected and characterized in different genotypes of wheat grains along maturation. [file mmc2.docx]

**Appendix 1.** Phenolic compounds detected and characterized in different genotypes of wheat grains along maturation with their molecular formula (MF), m/z, retention time (RT), score, fragmentation score (FS), mass error (ME) and isotope similarity (IS). Bold compounds mean those identified with following reference standards. Roman numerals (I, II, II, …) stand the respective isomers of the identified compounds.

|  | **Name** | **Formula** | **m/z** | **RT (min)** | **Score** | **FS** | **ME (ppm)** | **IS** |
| --- | --- | --- | --- | --- | --- | --- | --- | --- |
|  | ***PHENOLIC ACIDS*** |  |  |  |  |  |  |  |
| **1** | p-Coumaric acid | C_9_H_8_O_3_ | 163.04 | 0.96 | 39.5 | 15.3 | -7.96 | 91.15 |
| **2** | **4-Hydroxybenzoic acid** | **C_7_H_6_O_3_** | **137.02** | **2.27** | **51.8** | **69.7** | **-8.97** | **99.24** |
| **3** | *4-Hydroxybenzoic acid I* | C_7_H_6_O_3_ | 137.02 | 4.10 | 56.5 | 95.2 | -9.99 | 98.17 |
| **4** | 1-O-Sinapoyl-beta-D-glucose | C_17_H_22_O_10_ | 385.11 | 0.43 | 42.2 | 22.2 | 1.53 | 90.67 |
| **5** | 1-O-Sinapoyl-beta-D-glucose I | C_17_H_22_O_10_ | 385.11 | 3.15 | 37.2 | 1.43 | -3.44 | 88.69 |
| **6** | 3.4-Dihydroxy-5-methoxybenzoicacid I | C_8_H_8_O_5_ | 183.03 | 0.94 | 36.5 | ND | -8.91 | 92.47 |
| **7** | 3.4-Dihydroxy-5-methoxybenzoicacid II | C_8_H_8_O_5_ | 183.03 | 1.18 | 36.9 | ND | -7.15 | 92.64 |
| **8** | 3.4-Dihydroxy-5-methoxybenzoicacid III | C_8_H_8_O_5_ | 183.03 | 2.51 | 40.3 | 16.6 | -7.59 | 93.61 |
| **9** | 3-Feruloylquinic acid I | C_17_H_20_O_9_ | 367.10 | 0.44 | 43.9 | 26.6 | 2.51 | 95.81 |
| **10** | 3-Feruloylquinic acid III | C_17_H_20_O_9_ | 367.10 | 2.42 | 57.2 | 95.6 | -3.45 | 94.53 |
| **11** | 3-Feruloylquinic acid IV | C_17_H_20_O_9_ | 367.10 | 2.84 | 55.6 | 95.7 | -3.60 | 86.32 |
| **12** | 3-Feruloylquinic acid V | C_17_H_20_O_9_ | 367.10 | 2.95 | 57 | 95.9 | -3.01 | 92.80 |
| **13** | 3-Feruloylquinic acid VI | C_17_H_20_O_9_ | 367.10 | 3.22 | 55.3 | 98.2 | -2.60 | 81.33 |
| **14** | 3-O-Methylgallic acid | C_8_H_8_O_5_ | 183.03 | 3.23 | 36.7 | ND | -7.64 | 91.83 |
| **15** | *4-P-Coumaroylquinic acid* | C_16_H_18_O_8_ | 337.09 | 0.45 | 41.3 | 24.8 | 0.58 | 82.42 |
| **16** | 5-8'-Benzofuran dehydrodiferulic acid I | C_20_H_18_O_8_ | 385.09 | 3.82 | 43.4 | 22.1 | -3.02 | 98.46 |
| **17** | 5-8'-Benzofuran dehydrodiferulic acid II | C_20_H_18_O_8_ | 385.09 | 4.81 | 44.2 | 29.1 | -3.35 | 96.01 |
| **18** | 5-8'-Benzofuran dehydrodiferulic acid III | C_20_H_18_O_8_ | 385.09 | 5.61 | 39.7 | 7.63 | -3.27 | 94.76 |
| **19** | ***Caffeic acid*** | **C_9_H_8_O_4_** | **179.03** | **2.66** | **54.8** | **83.8** | **-7.50** | **98.69** |
| **20** | Caffeic acid ethyl ester I | C_11_H_12_O_4_ | 207.07 | 0.43 | 39.3 | 10.3 | 1.50 | 87.87 |
| **21** | Caffeic acid ethyl ester II | C_11_H_12_O_4_ | 207.06 | 5.12 | 38.1 | 9.47 | -6.88 | 88.67 |
| **22** | Caffeic acid I | C_9_H_8_O_4_ | 179.03 | 1.62 | 56.1 | 94.7 | -7.83 | 94.36 |
| **23** | Caffeic acid II | C_9_H_8_O_4_ | 179.03 | 1.93 | 51 | 74 | -8.43 | 90.52 |
| **24** | Caffeic acid III | C_9_H_8_O_4_ | 179.03 | 2.85 | 36.3 | ND | -7.92 | 90.56 |
| **25** | Caffeic acid IV | C_9_H_8_O_4_ | 179.03 | 3.43 | 36.5 | ND | -7.67 | 91.02 |
| **26** | Chlorogenic acid | C_16_H_18_O_9_ | 353.09 | 0.44 | 43.8 | 26.6 | 2.19 | 95.11 |
| **27** | *Cinnamic acid* | C_9_H_8_O_2_ | 147.04 | 3.82 | 47 | 54.7 | -9.68 | 90.68 |
| **28** | Diferulic acid I | C_20_H_18_O_8_ | 385.09 | 3.58 | 48.1 | 51 | -3.34 | 93.37 |
| **29** | Diferulic acid II | C_20_H_18_O_8_ | 385.09 | 3.74 | 43.3 | 24.2 | -3.26 | 96.00 |
| **30** | Diferulic acid III | C_20_H_18_O_8_ | 385.09 | 4.22 | 42.4 | 24.8 | -3.43 | 91.47 |
| **31** | Diferulic acid IV | C_20_H_18_O_8_ | 385.09 | 4.46 | 43.3 | 22.8 | -3.20 | 97.54 |
| **32** | Diferulic acid V | C_20_H_18_O_8_ | 385.09 | 4.89 | 43.5 | 38.7 | -3.28 | 82.51 |
| **33** | Diferulic acid VI | C_20_H_18_O_8_ | 385.09 | 5.24 | 40.4 | 23.5 | -3.05 | 81.93 |
| **34** | Dihydroconiferyl alcohol | C_10_H_14_O_3_ | 181.09 | 3.10 | 36.6 | ND | -7.01 | 90.81 |
| **35** | Ellagic acid | C_14_H_6_O_8_ | 301.00 | 3.48 | 36.4 | ND | -4.35 | 87.21 |
| **36** | ***Ferulic acid*** | **C_10_H_10_O_4_** | **193.05** | **3.52** | **43.7** | **26** | **-5.62** | **99.11** |
| **37** | Ferulic acid I | C_10_H_10_O_4_ | 193.05 | 2.15 | 54.4 | 90.1 | -7.27 | 89.85 |
| **38** | Ferulic acid II | C_10_H_10_O_4_ | 193.05 | 2.31 | 36.3 | ND | -6.98 | 89.46 |
| **39** | *Ferulic acid III* | C_10_H_10_O_4_ | 193.05 | 2.42 | 54.7 | 89.5 | -6.62 | 91.29 |
| **40** | Ferulic acid IV | C_10_H_10_O_4_ | 193.05 | 3.31 | 54.5 | 89.9 | -7.36 | 90.74 |
| **41** | *Ferulic acid V* | C_10_H_10_O_4_ | 193.05 | 3.68 | 49.9 | 57.4 | -6.16 | 99.21 |
| **42** | Ferulic acid VI | C_10_H_10_O_4_ | 193.05 | 4.46 | 44.3 | 40.4 | -6.69 | 88.84 |
| **43** | Ferulic acid VII | C_10_H_10_O_4_ | 193.05 | 4.81 | 51.6 | 67.6 | -7.05 | 98.31 |
| **44** | Ferulic acid VIII | C_10_H_10_O_4_ | 193.05 | 5.32 | 37.5 | ND | -8.10 | 96.48 |
| **45** | Feruloyl glucose I | C_16_H_20_O_9_ | 355.10 | 0.44 | 42 | 21.2 | -1.45 | 90.52 |
| **46** | Feruloyl glucose II | C_16_H_20_O_9_ | 355.10 | 2.15 | 56.1 | 96.5 | -3.10 | 87.49 |
| **47** | Feruloyl glucose III | C_16_H_20_O_9_ | 355.10 | 2.55 | 56.2 | 98.1 | -3.82 | 87.48 |
| **48** | Feruloyl glucose IV | C_16_H_20_O_9_ | 355.10 | 2.87 | 36.2 | ND | -3.26 | 84.91 |
| **49** | **Gallic acid** | **C_7_H_6_O_5_** | **169.01** | **1.22** | **37** | ND | **-8.16** | **93.95** |
| **50** | Gallic acid 3-O-gallate | C1_4_H_10_O_9_ | 321.02 | 1.98 | 36 | ND | -3.31 | 84.09 |
| **51** | Gallic acid ethyl ester | C_9_H_10_O_5_ | 197.04 | 1.59 | 56 | 96.1 | -7.66 | 92.70 |
| **52** | Gallic acid I | C_7_H_6_O_5_ | 169.01 | 3.24 | 55.4 | 93.9 | -8.54 | 92.46 |
| **53** | **Gentisic acid** | **C_7_H_6_O_4_** | **153.02** | **2.41** | **37.3** | ND | **-9.29** | **96.66** |
| **54** | Gentisic acid II | C_7_H_6_O_4_ | 153.02 | 1.59 | 55 | 86 | -8.97 | 99.07 |
| **55** | *Gentisic acid I* | C7H6O4 | 153.02 | 1.80 | 55.8 | 92.1 | -9.02 | 96.68 |
| **56** | *Gentisic acid III* | C7H6O4 | 153.02 | 2.80 | 47.2 | 46.8 | -8.79 | 98.82 |
| **57** | Gentisic acid IV | C7H6O4 | 153.02 | 3.24 | 40.1 | 11 | -9.05 | 99.31 |
| **58** | Gentisic acid V | C7H6O4 | 153.02 | 4.99 | 36.7 | ND | -9.06 | 93.53 |
| **59** | Methoxyphenylacetic acid | C_9_H_10_O_3_ | 165.05 | 4.33 | 38.6 | 11 | -8.10 | 91.23 |
| **60** | ***p-Coumaric acid*** | **C_9_H_8_O_3_** | **163.04** | **3.29** | **56.2** | **91.7** | **-8.50** | **98.98** |
| **61** | p-Coumaric acid 4-O-glucoside | C_15_H_18_O_8_ | 325.09 | 0.44 | 42.7 | 28.6 | -0.77 | 85.77 |
| **62** | p-Coumaric acid I | C_9_H_8_O_3_ | 163.04 | 3.12 | 36.5 | ND | -9.28 | 92.82 |
| **63** | p-Coumaric acid II | C_9_H_8_O_3_ | 163.04 | 3.45 | 36.9 | ND | -8.98 | 94.32 |
| **64** | p-Coumaric acid III | C_9_H_8_O_3_ | 163.04 | 4.31 | 39.7 | 17.7 | -9.39 | 91.28 |
| **65** | ***Sinapic acid*** | **C_11_H_12_O_5_** | **223.06** | **3.53** | **40.1** | **14.3** | **-6.29** | **93.26** |
| **66** | Sinapic acid I | C_11_H_12_O_5_ | 223.06 | 3.68 | 41.3 | 24.9 | -6.26 | 88.89 |
| **67** | Sinapic acid II | C_11_H_12_O_5_ | 223.06 | 4.53 | 36.7 | ND | -5.37 | 89.74 |
| **68** | Sinapic acid III | C_11_H_12_O_5_ | 223.06 | 4.62 | 36.3 | ND | -5.35 | 87.65 |
| **69** | **Syringic acid** | **C_9_H_10_O_5_** | **197.04** | **2.74** | **37** | ND | **-8.88** | **94.89** |
| **70** | Syringic acid I | C_9_H_10_O_5_ | 197.04 | 1.31 | 36.9 | ND | -6.93 | 92.09 |
| **71** | Syringin | C_17_H_24_O_9_ | 371.13 | 0.43 | 38.4 | 8.23 | -2.01 | 86.18 |
| **72** | Vanillic acid I | C_8_H_8_O_4_ | 167.03 | 1.60 | 56.6 | 95.2 | -8.17 | 96.77 |
| **73** | Vanillic acid II | C_8_H_8_O_4_ | 167.03 | 2.52 | 36.5 | ND | -8.24 | 91.49 |
| **74** | Vanillic acid III | C_8_H_8_O_4_ | 167.03 | 2.99 | 36.3 | ND | -8.18 | 90.85 |
| **75** | Vanillic acid IV | C_8_H_8_O_4_ | 167.03 | 3.24 | 54.2 | 88.2 | -7.15 | 90.85 |
| **76** | *Zingerone II* | C_11_H_14_O_3_ | 193.09 | 6.64 | 55.2 | 88.5 | -6.92 | 95.38 |
| **77** | Zingerone I | C_11_H_14_O_3_ | 193.09 | 4.60 | 37.7 | 8.99 | 8.95 | 89.40 |
|  | ***FLAVONOIDS*** |  |  |  |  |  |  |  |
| **78** | Apigenin | C_15_H_10_O_5_ | 269.04 | 5.60 | 35.9 | ND | -3.89 | 83.99 |
| **79** | (-)-Acanthocarpan | C_17_H_12_O_7_ | 327.05 | 4.66 | 35.7 | ND | -3.74 | 82.84 |
| **80** | (-)-Medicocarpin I | C_22_H_24_O_9_ | 431.13 | 4.36 | 35.7 | ND | -2.65 | 81.54 |
| **81** | (-)-Medicocarpin II | C_22_H_24_O_9_ | 431.13 | 4.94 | 49.3 | 68.9 | -2.66 | 80.85 |
| **82** | (-)-Medicocarpin III | C_22_H_24_O_9_ | 431.13 | 5.50 | 36.1 | ND | -2.48 | 83.31 |
| **83** | (3R)-Sativanone | C_17_H_16_O_5_ | 299.09 | 4.96 | 35.5 | ND | -4.13 | 82.15 |
| **84** | 3.5.7-Trimethoxyflavone | C_18_H_16_O_5_ | 311.09 | 4.58 | 43.1 | 38.8 | -4.09 | 81.27 |
| **85** | *3.7-Dimethylquercetin* | C_17_H_14_O_7_ | 329.07 | 5.70 | 35.9 | ND | -3.92 | 84.01 |
| **86** | 3.7-Dimethylquercetin I | C_17_H_14_O_7_ | 329.07 | 4.63 | 35.7 | ND | -2.98 | 81.78 |
| **87** | 3-O-Methylviolanone | C_18_H_18_O_6_ | 329.10 | 3.93 | 35.6 | ND | -4.11 | 82.76 |
| **88** | 5.7-Dimethoxyflavone I | C_17_H_14_O_4_ | 281.08 | 3.52 | 40.8 | 21.4 | -6.65 | 89.92 |
| **89** | 5.7-Dimethoxyflavone II | C_17_H_14_O_4_ | 281.08 | 3.74 | 46.9 | 47.1 | -5.24 | 93.56 |
| **90** | 5.7-Dimethoxyflavone III | C_17_H_14_O_4_ | 281.08 | 4.57 | 39.5 | 17.7 | -4.93 | 85.68 |
| **91** | 7.2'-Dihydroxy-4'-methoxyisoflavanone | C_16_H_14_O_5_ | 285.08 | 4.57 | 39.6 | 21.6 | -5.93 | 83.05 |
| **92** | 7-Hydroxyflavanone | C_15_H_12_O_3_ | 239.07 | 5.61 | 48.4 | 64.7 | -6.40 | 84.37 |
| **93** | Acerosin | C_18_H_16_O_8_ | 359.08 | 3.93 | 49.7 | 66.3 | 3.67 | 86.42 |
| **94** | Apigenin 6-C-glucoside | C_21_H_20_O_10_ | 431.10 | 5.60 | 37 | 0.94 | -2.74 | 87.31 |
| **95** | Apigenin 7-O-apiosyl-glucoside I | C_26_H_28_O_14_ | 563.14 | 2.87 | 57.4 | 96 | -1.25 | 92.45 |
| **96** | Apigenin 7-O-apiosyl-glucoside II | C_26_H_28_O_14_ | 563.14 | 3.00 | 54.4 | 80.4 | -1.25 | 93.14 |
| **97** | Apigenin 7-O-apiosyl-glucoside III | C_26_H_28_O_14_ | 563.14 | 3.12 | 36.7 | ND | -1.55 | 85.56 |
| **98** | Apigenin 7-O-glucoside | C_21_H_24_O_9_ | 419.13 | 3.21 | 53 | 77.4 | -4.84 | 92.99 |
| **99** | Apigenin 7-O-neohesperidoside | C_27_H_30_O_14_ | 577.16 | 3.71 | 56.7 | 98.8 | -1.14 | 85.90 |
| **100** | *Auriculoside I* | C_22_H_26_O_10_ | 449.15 | 6.29 | 35.8 | ND | 8.23 | 88.22 |
| **101** | *Auriculoside II* | C_22_H_26_O_10_ | 449.15 | 6.62 | 35.4 | ND | 8.27 | 86.34 |
| **102** | Carlinoside | C_26_H_28_O_15_ | 579.13 | 2.73 | 36.9 | ND | -1.31 | 85.93 |
| **103** | Cianidanol | C_15_H_16_O_7_ | 307.08 | 3.24 | 37.4 | ND | -2.09 | 89.38 |
| **104** | Dalbergin I | C_16_H_12_O_4_ | 267.06 | 3.59 | 50.3 | 72.1 | -5.52 | 85.55 |
| **105** | Dalbergin II | C_16_H_12_O_4_ | 267.06 | 3.82 | 51.9 | 78.6 | -5.65 | 87.20 |
| **106** | Dalpanin | C_26_H_30_O_12_ | 533.17 | 0.47 | 49.6 | 62.5 | 9.24 | 95.46 |
| **107** | Dasytrichone | C_18_H_16_O_4_ | 295.10 | 0.92 | 35.3 | ND | 8.90 | 86.13 |
| **108** | Dihydromyricetin I | C_15_H_12_O_8_ | 319.05 | 0.56 | 36.3 | 1.27 | 4.29 | 85.44 |
| **109** | Dihydroquercetin | C_15_H_12_O_7_ | 303.05 | 3.67 | 53.8 | 89.1 | -4.45 | 85.16 |
| **110** | Dihydroquercetin II | C_15_H_12_O_7_ | 303.05 | 0.55 | 37 | 0.78 | 2.60 | 87.14 |
| **111** | Eupatilin I | C_18_H_16_O_7_ | 343.08 | 4.12 | 36.2 | 1.9 | -3.48 | 83.03 |
| **112** | Eupatilin II | C_18_H_16_O_7_ | 343.08 | 4.75 | 47.3 | 49 | -3.69 | 91.93 |
| **113** | Eupatorin I | C_18_H_16_O_7_ | 343.08 | 5.05 | 35.6 | 1.45 | -3.66 | 80.88 |
| **114** | Eupatorin II | C_18_H_16_O_7_ | 343.08 | 5.31 | 39.6 | 11.3 | -3.68 | 91.22 |
| **115** | Formononetin | C_16_H_12_O_4_ | 267.06 | 5.61 | 48.8 | 59 | -5.35 | 91.14 |
| **116** | Gardenin B I | C_19_H_18_O_7_ | 357.10 | 4.16 | 47.5 | 58.6 | -3.29 | 82.54 |
| **117** | Gardenin B II | C_19_H_18_O_7_ | 357.10 | 4.44 | 35.6 | ND | -3.45 | 82.29 |
| **118** | Gardenin B III | C_19_H_18_O_7_ | 357.10 | 4.58 | 41.8 | 30.7 | -3.92 | 82.94 |
| **119** | Gardenin B IV | C_19_H_18_O_7_ | 357.10 | 4.84 | 35.3 | ND | -3.47 | 80.79 |
| **120** | Gardenin B V | C_19_H_18_O_7_ | 357.10 | 5.30 | 35.3 | 1.59 | -4.60 | 80.00 |
| **121** | Gardenin B VI | C_19_H_18_O_7_ | 357.10 | 5.73 | 43.5 | 36.5 | -4.14 | 85.86 |
| **122** | *Glycitin* | C_22_H_22_O_10_ | 445.11 | 3.46 | 51.3 | 71.3 | -2.25 | 88.08 |
| **123** | Glycyphyllin | C_21_H_24_O_9_ | 419.13 | 3.38 | 44.4 | 42.1 | -1.41 | 81.53 |
| **124** | Hesperetin I | C_16_H_14_O_6_ | 301.07 | 3.52 | 41.4 | 27.1 | -9.05 | 89.70 |
| **125** | *Hesperetin II* | C_16_H_14_O_6_ | 301.07 | 5.04 | 36.1 | ND | -3.96 | 85.27 |
| **126** | Hesperetin III | C_16_H_14_O_6_ | 301.07 | 5.33 | 35.5 | ND | -4.41 | 82.85 |
| **127** | **Kaempferol** | **C_15_H_10_O_6_** | **285.04** | **5.17** | **36.8** | ND | **-4.34** | **89.11** |
| **128** | Kievitone | C_20_H_20_O_6_ | 355.12 | 6.16 | 46.8 | 53 | -3.22 | 84.99 |
| **129** | Koparin | C_16_H_12_O_6_ | 299.05 | 5.75 | 36 | ND | -4.65 | 85.56 |
| **130** | Malvidin-3-(p-coumaroyl)-rutinoside-5-glucoside | C_44_H_51_O_23_ | 946.28 | 3.05 | 34.5 | ND | 9.73 | 83.29 |
| **131** | Myricetin I | C_15_H_10_O_8_ | 317.03 | 2.47 | 45.8 | 35.8 | 0.98 | 94.22 |
| **132** | Myricetin II | C_15_H_10_O_8_ | 317.03 | 2.97 | 54.3 | 89 | -2.12 | 85.29 |
| **133** | Naringenin | C_15_H_12_O_5_ | 271.06 | 4.64 | 39.7 | 19.1 | -4.57 | 84.65 |
| **134** | Neohesperidin | C_28_H_34_O_15_ | 609.19 | 0.48 | 44.4 | 45.9 | 8.42 | 85.25 |
| **135** | *Nobiletin I* | C_21_H_22_O_8_ | 401.12 | 3.46 | 35.9 | ND | -3.07 | 83.09 |
| **136** | *Nobiletin II* | C_21_H_22_O_8_ | 401.12 | 4.44 | 41.7 | 21.1 | -2.67 | 90.45 |
| **137** | Nobiletin III | C_21_H_22_O_8_ | 401.12 | 4.89 | 35.1 | ND | -5.17 | 81.62 |
| **138** | Pebrellin | C_19_H_18_O_8_ | 373.09 | 3.80 | 36.3 | ND | -3.14 | 85.28 |
| **139** | Phlorizin | C_21_H_24_O_10_ | 435.13 | 2.48 | 37.1 | ND | -1.52 | 87.55 |
| **140** | Pinobanksin 3-O-acetate | C_17_H_14_O_6_ | 313.07 | 5.80 | 35.5 | ND | -3.96 | 81.97 |
| **141** | Procyanidin dimer B1 I | C_30_H_26_O_12_ | 577.13 | 4.21 | 36.5 | 3.16 | -1.33 | 81.01 |
| **142** | Procyanidin dimer B1 II | C_30_H_26_O_12_ | 577.13 | 4.58 | 38.8 | 15.2 | -0.98 | 80.15 |
| **143** | Procyanidin dimer B1 III | C_30_H_26_O_12_ | 577.13 | 4.91 | 36.8 | 3.75 | -1.32 | 81.89 |
| **144** | Psoralidin | C_20_H_16_O_5_ | 335.09 | 1.01 | 35.3 | ND | -6.00 | 83.48 |
| **145** | Puerarin I | C_21_H_20_O_9_ | 415.10 | 4.44 | 37.8 | 9.55 | -2.88 | 83.09 |
| **146** | Puerarin II | C_21_H_20_O_9_ | 415.10 | 4.76 | 49.5 | 60.6 | -3.19 | 90.40 |
| **147** | Quercetin I | C_15_H_10_O_7_ | 301.03 | 1.88 | 50.1 | 69.9 | -4.98 | 86.14 |
| **148** | Quercetin II | C_15_H_10_O_7_ | 301.03 | 2.34 | 36.2 | ND | -3.36 | 85.09 |
| **149** | Quercetin III | C_15_H_10_O_7_ | 301.03 | 3.61 | 47.6 | 59.2 | -4.03 | 83.59 |
| **150** | Scutellarein | C_15_H_10_O_6_ | 285.04 | 3.73 | 46.9 | 50.9 | -2.40 | 86.45 |
| **151** | Sissotrin | C_22_H_22_O_10_ | 445.11 | 3.56 | 40.6 | 14.5 | -2.26 | 91.07 |
| **152** | Tectoridin | C_22_H_22_O_11_ | 461.11 | 3.22 | 36.2 | ND | -2.12 | 83.32 |
| **153** | Tephrowatsin A | C_22_H_26_O_4_ | 353.18 | 7.47 | 40.7 | 28.6 | 6.21 | 82.04 |
| **154** | Tetramethylscutellarein I | C_19_H_18_O_6_ | 341.10 | 3.58 | 43.6 | 27.3 | -3.52 | 94.63 |
| **155** | Tetramethylscutellarein II | C_19_H_18_O_6_ | 341.10 | 3.82 | 39.8 | 9.41 | -3.54 | 93.57 |
| **156** | Tetramethylscutellarein III | C_19_H_18_O_6_ | 341.10 | 4.38 | 37.3 | 7.67 | -3.72 | 83.07 |
| **157** | *Tetramethylscutellarein IV* | C_19_H_18_O_6_ | 341.10 | 4.57 | 39.5 | 15.8 | -3.22 | 85.55 |
| **158** | *Tetramethylscutellarein V* | C_19_H_18_O_6_ | 341.10 | 4.89 | 42.4 | 18.9 | -3.47 | 97.19 |
| **159** | Tetramethylscutellarein VI | C_19_H_18_O_6_ | 341.10 | 5.24 | 36.3 | 3.36 | -3.54 | 82.54 |
| **160** | Tetramethylscutellarein VII | C_19_H_18_O_6_ | 341.10 | 5.61 | 37.1 | 2.19 | -3.79 | 87.96 |
| **161** | Tetramethylscutellarein VIII | C_19_H_18_O_6_ | 341.10 | 5.89 | 36 | 0.019 | -3.46 | 84.03 |
| **162** | Vicenin-2 | C_27_H_30_O_15_ | 593.15 | 3.09 | 36.5 | ND | -1.00 | 83.92 |
|  | ***OTHER POLYPHENOLS*** |  |  |  |  |  |  |  |
| **163** | Coniferin | C_16_H_22_O_8_ | 341.12 | 2.13 | 36.5 | ND | -4.25 | 87.25 |
| **164** | 1.3.5-Trimethoxybenzene II | C_9_H_12_O_3_ | 167.07 | 2.00 | 36.3 | ND | -7.84 | 90.05 |
| **165** | 1.3.5-Trimethoxybenzene III | C_9_H_12_O_3_ | 167.07 | 2.38 | 36.3 | ND | -8.87 | 91.26 |
| **166** | 3.4-Dihydroxyphenylglycol I | C_8_H_10_O_4_ | 169.05 | 1.88 | 47.7 | 56.9 | -9.31 | 91.96 |
| **167** | 3.4-Dihydroxyphenylglycol II | C_8_H_10_O_4_ | 169.05 | 2.80 | 36.4 | ND | -8.86 | 91.82 |
| **168** | 3.4-Dihydroxyphenylglycol III | C_8_H_10_O_4_ | 169.05 | 2.94 | 36.2 | ND | -8.94 | 90.83 |
| **169** | 4-Vinylguaiacol I | C_9_H_10_O_2_ | 149.06 | 3.52 | 46.9 | 45.8 | -9.48 | 99.17 |
| **170** | 4-Vinylguaiacol II | C_9_H_10_O_2_ | 149.06 | 3.68 | 48.7 | 58.1 | -9.64 | 96.12 |
| **171** | 4-Vinylguaiacol III | C_9_H_10_O_2_ | 149.06 | 4.81 | 51.3 | 72.7 | -9.29 | 93.92 |
| **172** | 5.6.7-Trimethoxycoumarin I | C_12_H_12_O_5_ | 235.06 | 3.71 | 44.3 | 44.2 | 8.35 | 86.72 |
| **173** | 5.6.7-Trimethoxycoumarin II | C_12_H_12_O_5_ | 235.06 | 4.89 | 36 | ND | -5.85 | 86.72 |
| **174** | 5-Hydroxyferulic acid methyl ester I | C_10_H_10_O_5_ | 209.04 | 2.76 | 36.8 | ND | -7.13 | 91.83 |
| **175** | 5-Hydroxyferulic acid methyl ester II | C_10_H_10_O_5_ | 209.04 | 3.08 | 53.8 | 86.7 | -6.65 | 89.73 |
| **176** | 5-Hydroxyferulic acid methyl ester III | C_10_H_10_O_5_ | 209.04 | 4.45 | 38.4 | 6.61 | -6.42 | 92.46 |
| **177** | Arbutinum | C_12_H_16_O_7_ | 271.08 | 0.99 | 38.3 | 4.55 | -4.22 | 91.69 |
| **178** | Byakangelicin | C_12_H_16_O_7_ | 333.10 | 3.47 | 36.7 | ND | -4.28 | 88.54 |
| **179** | Elemicin I | C_12_H_16_O_3_ | 207.10 | 4.72 | 36.3 | ND | -4.91 | 87.10 |
| **180** | *Elemicin II* | C_12_H_16_O_3_ | 207.10 | 6.55 | 53.1 | 82.1 | -6.84 | 91.10 |
| **181** | Elemicin III | C_12_H_16_O_3_ | 207.10 | 7.48 | 36.2 | 2.26 | -7.85 | 87.66 |
| **182** | Esculetin I | C_9_H_6_O_4_ | 177.02 | 2.21 | 36.6 | ND | -7.95 | 91.68 |
| **183** | *Esculetin II* | C_9_H_6_O_4_ | 177.02 | 2.62 | 54.1 | 84.7 | -7.77 | 94.26 |
| **184** | Esculetin III | C_9_H_6_O_4_ | 177.02 | 2.40 | 36.4 | ND | -7.86 | 90.86 |
| **185** | Esculetin IV | C_9_H_6_O_4_ | 177.02 | 2.84 | 36.4 | ND | -7.70 | 90.69 |
| **186** | Esculetin V | C_9_H_6_O_4_ | 177.02 | 3.13 | 36.8 | ND | -7.91 | 92.91 |
| **187** | Esculetin VI | C_9_H_6_O_4_ | 177.02 | 3.31 | 53.3 | 83.4 | -7.71 | 91.44 |
| **188** | EsculetinVII | C_9_H_6_O_4_ | 177.02 | 3.69 | 43.2 | 34.7 | -7.73 | 89.91 |
| **189** | Esculin | C_15_H_16_O_9_ | 339.07 | 0.44 | 39.2 | 17 | 2.38 | 81.63 |
| **190** | Ferulaldehyde I | C_10_H_10_O_3_ | 177.05 | 3.40 | 36.4 | ND | -6.94 | 90.00 |
| **191** | Ferulaldehyde II | C_10_H_10_O_3_ | 177.05 | 5.16 | 55.1 | 89.1 | -7.73 | 94.96 |
| **192** | Fraxetin | C_10_H_8_O_5_ | 207.03 | 2.77 | 48.1 | 56.7 | -7.08 | 91.84 |
| **193** | Hydroxytyrosol | C_8_H_10_O_3_ | 153.05 | 1.08 | 36.6 | ND | -9.48 | 93.62 |
| **194** | Juglone | C_10_H_6_O_3_ | 173.02 | 4.18 | 36.6 | ND | -7.25 | 91.24 |
| **195** | Leptodactylone I | C_11_H_10_O_5_ | 221.04 | 3.57 | 38.3 | ND | -5.31 | 97.56 |
| **196** | Leptodactylone II | C_11_H_10_O_5_ | 221.04 | 4.17 | 37 | ND | -5.71 | 91.58 |
| **197** | Leptodactylone III | C_11_H_10_O_5_ | 221.04 | 4.30 | 48.1 | 57 | -6.40 | 91.00 |
| **198** | Myristicin | C_11_H_12_O_3_ | 191.07 | 6.02 | 54.4 | 91.5 | -6.71 | 88.15 |
| **199** | Oleoside Dimethylester | C_18_H_26_O_11_ | 417.14 | 1.29 | 36.1 | ND | -2.40 | 83.50 |
| **200** | Scopoletin I | C_10_H_8_O_4_ | 191.03 | 3.52 | 41.3 | 25.5 | -7.04 | 88.86 |
| **201** | Scopoletin II | C_10_H_8_O_4_ | 191.03 | 5.22 | 36.2 | ND | -7.10 | 88.86 |
| **202** | Sinapaldehyde I | C_11_H_12_O_4_ | 207.07 | 3.26 | 36.5 | ND | -5.69 | 89.07 |
| **203** | Sinapaldehyde II | C_11_H_12_O_4_ | 207.06 | 3.39 | 36 | ND | -7.10 | 87.87 |
| **204** | Syringaldehyde I | C_9_H_10_O_4_ | 181.05 | 2.01 | 36.5 | ND | -7.41 | 90.84 |
| **205** | Syringaldehyde II | C_9_H_10_O_4_ | 181.05 | 2.10 | 36.6 | ND | -7.44 | 91.57 |
| **206** | Syringaldehyde III | C_9_H_10_O_4_ | 181.05 | 3.14 | 36.4 | ND | -8.22 | 91.00 |
| **207** | Syringaldehyde IV | C_9_H_10_O_4_ | 181.05 | 3.40 | 36.2 | ND | -8.56 | 90.70 |
| **208** | Syringaldehyde V | C_9_H_10_O_4_ | 181.05 | 4.24 | 36.4 | ND | -8.13 | 90.91 |
| **209** | Vanillactic Acid I | C_10_H_12_O_5_ | 211.06 | 1.90 | 52.7 | 77.4 | -6.79 | 93.85 |
| **210** | Vanillactic Acid II | C_10_H_12_O_5_ | 211.06 | 2.00 | 36.3 | ND | -6.34 | 88.60 |
| **211** | Vanillactic Acid III | C_10_H_12_O_5_ | 211.06 | 2.30 | 36.1 | ND | -7.28 | 88.60 |
| **212** | Vanillin I | C_8_H_8_O_3_ | 151.04 | 1.90 | 52.8 | 81.8 | -9.69 | 92.98 |
| **213** | **Vanillin** | **C_8_H_8_O_3_** | **151.04** | **2.04** | **34.4** | **1.04** | **-8.53** | **80.35** |
|  | ***LIGNANS*** |  |  |  |  |  |  |  |
| **214** | 4'-Demethyldeoxypodophyllotoxin | C_21_H_20_O_7_ | 383.11 | 0.91 | 37.9 | ND | -0.50 | 90.22 |
| **215** | beta-Peltatin A methyl ether | C_23_H_24_O_8_ | 427.14 | 5.51 | 37.6 | 2.07 | 3.09 | 89.44 |
| **216** | Cleistanthin A | C_28_H_28_O_11_ | 539.16 | 1.33 | 35 | ND | 9.87 | 85.54 |
| **217** | Diphyllin | C_21_H_16_O_7_ | 379.08 | 0.43 | 39.5 | 5.5 | -0.03 | 91.94 |
| **218** | Flaxseed I | C_26_H_38_O_12_ | 541.23 | 2.19 | 37.4 | ND | 1.25 | 88.60 |
| **219** | Flaxseed II | C_26_H_38_O_12_ | 541.23 | 2.55 | 36.8 | ND | 0.88 | 84.99 |
| **220** | *Magnoshinin* | C_24_H_30_O_6_ | 413.19 | 5.88 | 36.7 | 2.04 | -9.79 | 92.22 |
| **221** | Matairesinol | C_20_H_22_O_6_ | 357.13 | 4.45 | 42.2 | 32.5 | -4.19 | 83.34 |
| **222** | Nortrachelogenin | C_20_H_22_O_7_ | 373.13 | 4.58 | 48.5 | 51.8 | -3.49 | 94.67 |
| **223** | Podophyllotoxin I | C_22_H_22_O_8_ | 413.12 | 4.94 | 35.6 | ND | -3.01 | 81.63 |
| **224** | Podophyllotoxin II | C_22_H_22_O_8_ | 413.12 | 6.46 | 36.3 | ND | -3.22 | 85.10 |
| **225** | Podorhizol beta-D-glucoside | C_28_H_34_O_13_ | 577.20 | 0.97 | 37 | 8.85 | 9.31 | 86.59 |
| **226** | Schisandrin A | C_24_H_32_O_6_ | 415.21 | 5.79 | 35.2 | ND | -9.23 | 86.12 |
| **227** | Secoisolariciresinol | C_20_H_26_O_6_ | 361.17 | 4.71 | 37.4 | ND | -1.65 | 88.77 |
| **228** | Sesamolin | C_20_H_18_O_7_ | 369.10 | 0.63 | 36 | ND | -0.82 | 80.93 |
| **229** | Sesamolinol | C_20_H_20_O_7_ | 371.11 | 4.69 | 44 | 44.3 | -3.84 | 80.35 |
|  | ***STILBENES*** |  |  |  |  |  |  |  |
| **230** | 3-[2-(2-Hydroxyphenyl)ethyl]-5-methoxyphenol | C_15_H_16_O_3_ | 243.10 | 3.52 | 37.2 | 3.18 | 2.38 | 85.62 |
| **231** | Batatasin I | C_17_H_16_O_4_ | 283.10 | 4.91 | 36.8 | ND | -5.27 | 89.93 |
| **232** | Chlorophorin | C_24_H_28_O_4_ | 379.19 | 4.68 | 34.5 | 0.09 | -9.23 | 82.43 |
| **233** | *Resveratrol 3-O-glucoside I* | C_20_H_22_O_8_ | 389.12 | 3.19 | 36.9 | ND | -2.47 | 87.32 |
| **234** | Resveratrol 3-O-glucoside II | C_20_H_22_O_8_ | 389.12 | 3.48 | 36.6 | ND | -3.25 | 86.67 |
| **235** | Resveratrol 3-O-glucoside III | C_20_H_22_O_8_ | 389.12 | 3.56 | 36.8 | ND | -3.20 | 87.94 |
| **236** | Resveratrol 3-O-glucoside IV | C_20_H_22_O_8_ | 389.12 | 3.66 | 35.9 | ND | -3.58 | 83.76 |
| **237** | Resveratrol 3-O-glucoside V | C_20_H_22_O_8_ | 389.12 | 3.85 | 37.7 | 10.4 | -3.18 | 81.88 |
